# Updated general exposure factors for risk assessment in the Korean population

**DOI:** 10.1038/s41370-022-00437-6

**Published:** 2022-04-14

**Authors:** Hyojung Yoon, Jungkwan Seo, Sun-Kyoung Yoo, Pil-Je Kim, Jinhyeon Park, Youngtae Choe, Wonho Yang

**Affiliations:** 1 Environmental Health Research Division, National Institute of Environmental Research, Incheon, Republic of Korea; 2 School of Environmental Engineering, University of Seoul, Seoul, Republic of Korea; 3 Department of Occupational Health, Daegu Catholic University, Gyeongbuk, Republic of Korea

**Keywords:** Exposure factors, Risk assessment, Anthropometric parameters, Inhalation rates, Food and drinking water intake, Time-activity patterns

## Abstract

**Background:**

There has been an increasing need to update the recommended values of Korean exposure factors for adults aged 19 and older, as using exposure factors developed over a decade ago could reduce risk assessment reliability.

**Objective:**

Exposure factor data have been compiled and standardized using the latest national statistical reports and academic literature, as well as studies conducted from 2016 to 2018.

**Methods:**

The updated data contained anthropometric parameters, inhalation rates, food and drinking water ingestion rates, and time-activity patterns and provided technical information on Koreans’ exposure factors classified by sex, age group, per capita and general population, and doer-only for various exposure assessments.

**Results:**

Although the average life expectancy, body weight, body surface area, and inhalation rate increased slightly compared to the 2007 Korean Exposure Factor Handbook, differences various in food consumption were remarkable. Because of Asians’ similar food preferences, the intake rate of grain products and vegetables in Koreans, Chinese, and Japanese contributed much toward total intake. Koreans spent half their times outdoors compared to Americans and Chinese.

**Significance:**

This study provided the currently updated exposure factor information for Koreans and could be compared with recommendations provided by exposure factor resources in various countries.

**Impact statement:**

- Exposure to environmental pollutants may significantly vary depending on the exposure factors related to human behaviors and characteristics. Therefore the exposure factors need to be continuously updated along with more extensive survey areas and improved measurement methods. We utilized the existing data with the aim to develop general exposure factors for risk assessment in Korean aged ≥19 years. Measurements and questionnaire surveys were also performed if there were no existing data. This study provided the currently updated exposure factor information for Koreans and could be compared to those of other countries.

## Introduction

Evaluating human exposure to environmental pollutants or stressors is an essential part of risk assessment. The traditional exposure assessment method indirectly estimates the exposure for a specific population group by applying the chemical concentration and time spent in microenvironments to a mathematical exposure model [1]. Exposure factors are parameters related to human anthropometric data and behaviors such as inhalation rate, consumption, exposure duration and frequency, and patterns of use of consumer products required to assess exposure to an individual or population [2, 3]. These exposure factors may be underestimated or overestimated in exposure and risk assessment owing to the application of exposure factor data from other countries. If specific population characteristics because of demographic, racial, cultural, and social differences are not properly reflected, the reliability of exposure assessment may decrease. Therefore, several countries have participated in efforts to compile and standardize human exposure factors to perform reliable risk assessments [4].

Exposure factor resources have been developed in Europe [5–7], the United States [2], Canada [8], Australia [9], China [10], Japan [11], and Korea [12, 13]. Synchronizing and integrating exposure factors across countries is necessary to develop standardized methodologies and protocols for collecting and analyzing data while respecting the inherent variability of exposure factors and characterizing the variability and uncertainty of these data [14]. The “Korean Exposure Factors Handbook” was published by the Ministry of Environment in 2007 [12]; thus, the recommended values of exposure factors should be updated.

This study aims to summarize the currently updated exposure factor information for Koreans and provide useful exposure factor resources for the contaminants exposure assessment. The results of exposure factors for Koreans could be compared to those of other countries and ultimately used for exposure and risk assessment. We have provided recommendations that can be found in each source to help understand racial and geographical characteristics, although there are differences between countries in study methodologies, the set of age groups, and statistical measures used.

## Methods

In this study, the general exposure factors of Koreans were divided into four categories, and the recommended values were calculated, including physiological parameters, inhalation rates, food and drinking water intake, and time-activity patterns for adults aged 19 years and older. The latest data from the Korea National Statistical Office (KOSTAT), Korea National Health and Nutrition Examination Survey (KNHANES), and other studies were used to develop the general exposure factors. Measurements and questionnaire surveys were also performed if there were no existing data. The recommended values for exposure factors were computed based on pooled data, which were collected from official and population-based Korean data. The screening criteria and principles for the studies we selected were as follows. In order to collect and standardize human exposure factors, (1) data with a sample size that can represent the country, (2) through reliable research institutes are required. (3) Data from studies conducted annually or periodically take precedence over those from temporary investigations. In other words, the data used to calculate the recommended values may be able to identify patterns of changes in exposure factors in Koreans over time. For example, the KNHANES is an annual survey conducted by stratifying samples by administrative district and socioeconomic status to consider national representativeness. These data on weight/height and food intake in 2010–2016 have sample sizes corresponding to 0.014% and 0.010% of the total population, respectively. The Time-Use Survey is conducted every five years by KOSTAT, and 0.055% of the total population was surveyed in 2014. This study was approved by the Institutional Review Board (CUIRB-2016–0104) at Daegu Catholic University.

### Anthropometric parameters

We used the latest data reported annually by the KOSTAT to identify life expectancy by gender and age [15]. Body weight and body surface area (BSA), defined as the total area of human skin surface, were computed by gender and age using data from the KNHANES 2013–2016 [16]. The weighted average and standard deviation for body weight and BSA were calculated by applying the sample size weights by the survey period of the KNHANES data. We used a regression equation formulated based on actual measurements of 34 males and 31 females (ages 20 to 62 years) reported by Lee et al. [17]. The coefficient of determination (*r*^2^) of the multiple linear regression models was >0.96. The formula for both sexes combined was finally selected because there was no significant difference according to sex or body type and the mean error was −0.1% [17]. The recommended BSA values were derived for both sexes using their average weights (W) and heights (H) in the following equation:

$${{{{{\mathrm{BSA}}}}}}\left( {{{{{{\mathrm{cm}}}}}}^2} \right) \,=\, 0.007331 \,\ast\, {{{{{\mathrm{W}}}}}}^{0.425} \,\ast\, {{{{{\mathrm{H}}}}}}^{0.725}$$

The body surface area for each body part was used as a percentage of the total BSA with a head of 7.6%, torso (including the neck) 37.4%, arms 14.8%, hands 4.8%, legs 28.9%, and feet 6.5% [18]. The relative proportions of each body part are listed in Table S1.

### Inhalation rate

Inhalation rates are required to assess the level of air pollutant exposure through inhalation. Adults aged 19 to 64 years were categorized into six age groups at 10-year intervals. The 19–24, 25–34, 35–44, 45–54, and 55–64 age groups, respectively, consisted of 20 participants for each gender. Participants were selected from non-smokers with no health problems, such as obesity, high blood pressure, and diabetes. A gas analyzer (Quark b2, COSMED, Italy) was used to measure the maximal oxygen uptake (VO_2_max), maximal ventilation (VEmax), maximal heart rate (HRmax), and ventilation threshold (VT) at different activity phases such as resting (in a sedentary state), walking (at 3.5–4.0 km/h), and slow running (at 5.5–6.0 km/h) for 10 min, respectively. These data were used to calculate minute ventilation (VE) which was defined as the short-term inhalation rate with the amount of air inhaled per minute for each activity phase [19]. The VE during sleep for Koreans was estimated by applying the ratio of VE during sleep to VE at rest, as reported by the US Environmental Protection Agency (EPA) [20].

The average daily inhalation rate is calculated by summing up the outcome of VE (L/min) and time spent (min/day) at various levels of physical activity [21]. The method of time-weighted daily inhalation rate was estimated from measurements of short-term inhalation rates during sleeping, resting, walking, and running slowly and time spent per day at those activity levels. Physical activities of 194 participants were recorded every 10 min in the time-activity diary for three consecutive days. According to KOSTAT’s classification criteria of 144 activity patterns [22], the activity diary for 3 days was divided into 4 levels: sleep, resting, walking, and slow running, and then the time spent per day was calculated for each activity. The daily inhalation rate (m^3^/day and m^3^/kg/day) was calculated by applying the participant’s measured VEs, time spent at each activity level, and body weight.

### Food and drinking water intake rates

Five thousand four hundred and twenty-two foods were classified into 14 food groups and 150 subgroups using the data from KNHANES to 2010–2015 [16] with detailed classification for each food group given in Table S2. Reflecting the characteristics of Korean consumption, average and percentile values were presented for each food intake, such as grains, vegetables, fruits, meat, eggs, fish and seafood, milk and dairy products, sugars, and fabricated foods. For the data on 31,418 people for food and 31,379 people for drinking water, we calculated both the per capita intake and the consumer-only intake for those who consumed these foods. To adjust the amount of water loss according to various cooking methods, different consumption calculation standards were applied for each food group consumed as raw and cooked ingredients [23]. This study reclassified food groups according to the criteria used in previous studies [2]. Fabricated foods, consisting of several food groups, applied their contributions to each food group based on weight, and the contribution rate was calculated using the recipe database provided by the Korean Nutrition Society [24].

### Time-activity patterns

The 2014 time-use survey data from the KOSTAT [22] were used to develop the exposure factors related to the time-activity patterns, which were recorded by the participants for 24 h a day at 10 min intervals by extracting a representative sample from all Koreans. These data were representative and reliable, as they surveyed the time spent on each activity by adults aged 19 years or older out of approximately 28,000 on weekdays (13,018 males, 15,134 females) and weekends (8706 males, 10,094 females) over a 5-year period. However, it is unclear whether the activity and action spaces were indoors or outdoors because of differences in the investigation’s purpose. Therefore, another small-scale survey in this study was also conducted on the activity patterns of 2526 adult males and females (1276 in metropolitan cities and 1250 in small and medium-sized cities) at 10 min intervals on 2 consecutive weekdays and weekends to calculate the indoor/outdoor ratio of the activity spaces.

Stratified sampling according to a square root proportional distribution was used nationwide for participant selection, and the male-to-female ratio was ~50:50. The space of activity was classified into indoor, outdoor, and in transit. Indoor spaces included homes, offices, schools, restaurants, pubs, shopping malls, markets, cultural facilities, fitness rooms, and other places. Transportation used in daily life included bicycles, private cars, public transport (buses, trains, etc.), and others. We reclassified time-activity patterns by applying the indoor and outdoor time ratios surveyed in this study to the time-use survey data from KOSTAT. The mean and percentiles for each recommended value were calculated by dividing the time spent by place, on each activity, and in transit.

## Results

### Anthropometric factors

**Fig. 1:**
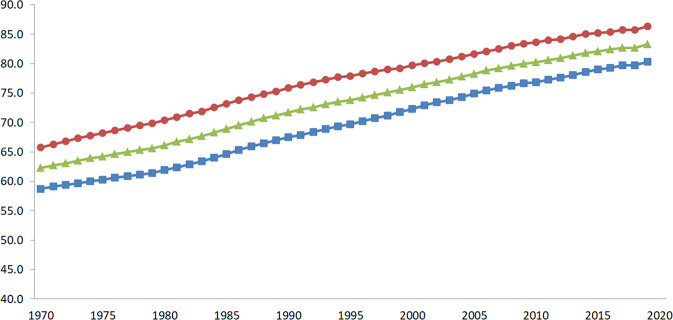
Life expectancy trends in Korea from 1970 to 2019 adapted from the KOSIS (2021). The figure is a line graph showing the life expectancy (age) of Koreans from 1970 to 2019, with blue squares () for men, red circles () for women, and green triangles () for both sexes combined. X-axis: Year, Y-axis: Life expectancy (age).

The life expectancy at birth for the Korean population was 83.3 years (79.7 and 85.7 years for males and females, respectively) in 2019. From 1970 to 2019, life expectancy increased by ~21 years over the past decades as shown in Fig. 1. The average body weight of Korean adults was 64.5 kg, with 71.5 kg for males and 57.7 kg for females (Table S3). Males’ weight showed the highest values in the 25–34 age group and then gradually decreased, while females’ weight peaked in the 45–54 age group. The BSA of Korean adults, calculated using body weight and height, was 18,670 cm^2^ for males and 16,058 cm^2^ for females (Table S4). In addition, the average and percentile of detailed BSA for each body part calculated from the proportion for each body part are presented in Table S5.

### Inhalation rates

Six out of 200 participants were excluded from the time-activity diary that was inappropriate. The VE according to the different activity levels (rest, walking, and slow running) for 194 participants in the laboratory is presented in Table S6. Both short-term inhalation rates for males and females increased with increased physical activity levels. Table S7 shows the daily inhalation rate by applying VE for each activity level to the time-activity patterns for 24 h. The recommended values for the daily inhalation rate for males and females were 16.2 and 13.0 m^3^/day, respectively.

### Food and drinking water intake rates

The per capita food consumption was relatively high, with grains, vegetables, fruits, and meat products at 600.23, 425.82, 187.01, and 116.69 g/day, respectively (Table 1). In addition, beverages and drinking water were 276.40 and 1008.33 mL/day, respectively. Among these food groups, grains, vegetables, and drinking water had the highest frequency of 99.6%, whereas fabricated foods (6.0%), milk and dairy products (38.0%), and seaweeds (49.1%) showed a lower intake frequency. The doer intakes of fruits, milk and dairy products, and fabricated foods were 284.08, 215.70, and 123.20 g/day, respectively, indicating a difference of 97.07, 130.64, and 114.28 g/day from the per capita food intakes as shown in Table S8.

**Table 1:** Per capita food and drinking water intake rates (g/day).

Food groups		*N*	Frequency (%)	Mean^a^	S.D.^b^	25th	50th	75th	95th
Grains	Foods	31,418	99.6	600.23	317.96	384.69	558.37	760.55	1160.63
	Ingredients	31,418	99.4	240.59	126.99	155.83	226.05	307.22	454.81
Vegetables		31,418	99.6	425.82	300.62	225.09	364.39	551.26	964.29
Fruits		31,418	67.9	187.01	301.11	0.00	68.08	265.70	748.90
Meat products		31,418	69.9	116.69	219.20	0.00	49.33	141.62	439.26
Eggs		31,418	50.4	27.08	45.82	0.00	4.77	39.22	117.26
Fish and seafood		31,418	84.7	84.50	163.42	3.25	30.34	102.31	342.49
Nuts and seeds products		31,418	75.9	6.49	35.58	0.02	0.49	2.34	29.35
Seaweeds	Raw	31,418	49.1	22.97	65.86	0.00	0.00	28.36	97.48
	Dried	31,418	49.1	2.71	9.44	0.00	0.00	3.17	11.30
Milk and dairy products		31,418	38.0	85.06	153.83	0.00	0.00	138.45	410.13
Fats and oils		31,418	89.7	8.73	11.61	1.31	5.14	12.04	29.81
Beverages^b^		31,418	81.1	276.40	547.95	3.62	36.79	350.64	1190.03
Seasonings		31,418	98.4	61.53	75.41	18.78	42.05	78.88	179.36
Sugars and sweeteners		31,418	77.8	10.18	19.95	0.30	3.74	12.18	40.75
Fabricated foods		31,418	6.0	8.92	45.35	0.00	0.00	0.00	47.13
Water^c^		31,379	99.6	1008.33	657.05	600	1000	1200	2000

^a^Arithmetic mean.

^b^*S.D.* standard deviation.

^c^Beverages and drinking water were calculated in mL/day.

### Time-activity factors

The time spent indoors for 24 h on weekdays was on average 15.10, 3.83, and 0.18 h at home, work, and school, respectively (Table 2). In other indoor areas such as restaurants, bars, shopping malls, markets, cultural facilities, and gyms, 1.65 h was spent. The time spent outdoors and in transit, including bicycles, for Koreans was 2.07 h and 1.17 h per day, respectively. On weekends, the time spent at home and in other indoor and outdoor spaces was 17.00 h, 1.88 h, and 2.34 h, respectively, which was higher than the time spent on weekdays. The time spent indoors, outdoors, and transportation by doers, which indicate the respondents who reported participating in each activity category, is shown in Table S9. Females spent 3.15 and 1.99 h more at home than males on weekdays and weekends, whereas males spent more time at offices and outdoors at 1.62 and 0.58 h on weekdays and 0.58 and 1.29 h on weekends, respectively Table S10).

**Table 2:** Time spent indoors, outdoors, and during transportation (h/day).

Category	Location		Mean^a^	S.D.^b^	25th	50th	75th	95th
Weekdays	Indoors	Home	15.10	4.76	11.49	14.15	19.14	23.13
		Workplace	3.83	4.35	0.00	0.63	8.17	11.02
		School	0.18	1.10	0.00	0.00	0.00	0.00
		Others	1.65	1.89	0.34	1.08	2.35	5.15
	Outdoors		2.07	1.93	0.78	1.52	2.73	5.80
	Transport		1.17	1.19	0.00	1.00	1.67	3.33
Weekends	Indoors	Home	17.00	4.72	13.49	17.60	20.81	23.92
		Workplace	1.60	3.34	0.00	0.00	0.00	9.54
		School	0.03	0.38	0.00	0.00	0.00	0.00
		Others	1.88	1.87	0.49	1.39	2.73	5.49
	Outdoors		2.34	2.12	0.92	1.83	3.15	6.50
	Transport		1.16	1.33	0.00	0.83	1.67	3.67
Both	Indoors	Home	15.86	4.83	12.00	15.67	19.98	23.49
		Workplace	2.94	4.13	0.00	0.00	6.95	10.67
		School	0.12	0.89	0.00	0.00	0.00	0.00
		Others	1.74	1.89	0.40	1.20	2.51	5.30
	Outdoors		2.18	2.01	0.83	1.64	2.91	6.09
	Transport		1.16	1.25	0.00	1.00	1.67	3.50

^a^Arithmetic mean.

^b^*S.D.* standard deviation.

## Discussion

### Comparison with Korean Exposure Factor Handbook in 2007

According to a study by Jang et al. [25] using national statistical data from 2003 to 2004, the average weight of males and females in Korea was calculated to be 68.5 and 53.9 kg, which meant that the body weight of Korean adults increased by about 2.3 kg for males and 1.3 kg for females over about 10 years. The average total BSA calculated from the weight and height data [16] was 17,804 cm^2^ with the feet 1162 cm^2^ and hands 837 cm^2^. In the past decade, the BSA has increased as well as weight gain [25]. Grasgruber et al. [26] analyzed the correlation between male height and socioeconomic and nutritional variables in 105 countries, and found that the human development index calculated by the United Nations and protein consumption were highly association with height. High life expectancy, GDP per capita, protein intake of milk, dairy products, eggs, and red meat had the most positive effect on height increase. This was consistent with our results, and the increase in Korean height compared to 10 years ago was accompanied by a gain in body weight and BSA. The long-term inhalation rates for Korean adults aged 18 to 49 years were 15.7 m^3^/day for males and 12.8 m^3^/day for females [25], similar to the values in this study.

For food and drinking water consumption, compared to previous studies in Koreans [23, 25], grain, seaweed, and drinking water intake rates decreased by 26.2%, 33.3%, and 32.9%, respectively, whereas intake rates of meat products, milk and dairy products, seasonings, eggs, and nut and seed products increased by 1.6–2.0 times. Traditional Korean diets with carbohydrates-containing foods such as white rice, noodle, and other grain as the main energy source were associated with lowering high density lipoprotein (HDL)-cholesterol [27]. For this reason, a diet low in carbohydrates and high in animal fat was recommended by modifying the acceptable macronutrient distribution ranges for carbohydrate and total fat intake from 55–70 to 55–65% and from 15–25 to 15–30%, respectively [28]. In addition, as the diet has been diversified compared to 10 years ago, the consumption of animal-sourced foods such as meat, eggs, milk, and dairy products has steadily been increasing, and Koreans eating habits are becoming westernized. In the previous literature that uses the 2004 time-use survey data [29], the time spent by Korean adults indoors, outdoors, and vehicles was 21.35, 1.27, and 1.38 h/day, respectively. Compared with a decade ago, the time spent outdoors was longer (0.91 h/day) and the time spent indoors and in transit was less with 0.69 and 0.22 h/day, respectively.

### Comparison with overseas data

Exposure factor data have been published in the form of handbooks, guidelines, and databases in many countries such as the United States, Canada, Europe, Australia, China, and Japan to collect, develop, standardize, and update various exposure factors required for human exposure assessment. Exposure factors affected by physiological, cultural, racial, and demographic characteristics may differ among countries, and it could be difficult to directly compare exposure factors due to the various survey periods, methodologies, and publication years of resources. However, the comparison of exposure factors from national resources is considered to be important because exposure factors of their own country or neighboring countries are used in the regional exposure assessment for chemical substances and stressors. The life expectancy, body weight, BSA, and inhalation rate from general exposure factor data in Korea and other countries were compared, as shown in Table 3. The average life expectancy of Koreans was higher than the recommended values in the exposure factor data for China, Japan, Australia, and the United States [2, 7, 9–11]. According to the latest data from the World Health Organization [30], Japan had the highest life expectancy in 2019 was Japan (84.26 years), followed by Switzerland (83.45 years) and South Korea (83.30 years). Koreans had low mortality rates from heart attacks, ischemic heart disease, and cancer, and had the second lowest rate of overweight and obesity (33.7%) after Japanese. These reasons might have contributed to improving the life expectancy of Koreans [31].

**Table 3:** Recommended values of physiological parameters and inhalation rates in Korean and other countries’ exposure factor data.

Exposure factors	Sex	Korea	China	Japan	Australia	Europe^a^	US
Life expectancy (years)	Male	79.7	72.38	77.72	79.3	–	75
	Female	85.7	77.37	84.6	83.9	–	80
	Both	82.7	74.83	–	81.6	–	78
Body weight (kg)	Male	71.5	66.1	64	85	70	–
	Female	57.7	57.8	52.7	70	60	–
	Both	64.5	61.4	–	78	–	80
BSA (m^2^)	Male	1.87	1.7	1.69	2.1	2.03	2.06
	Female	1.61	1.5	1.51	1.9	1.77	1.85
	Both	1.74	1.6	–	2	–	–
Inhalation rate (m^3^/day)	Male	16.21	17.7	–	–	15.2	–
	Female	13.03	14.5	–	–	11.3	–
	Both	14.61	16.1	17.3	15	–	12.2–16.0^b^

^a^European data on Body weight (Nordic), BSA (Sweden, ECTEC, 2001), Inhalation rate (ECTEC, 2001).

^b^Range of inhalation rates for US adults 21 years and over (21 to <31 yrs, 15.7 m^3^/day; 31 to <41 yrs, 16.0 m^3^/day; 41 to <51 yrs, 16.0 m^3^/day; 51 to <61 yrs, 15.7 m^3^/day; 61 to <71 yrs, 14.2 m^3^/day; 71 to <81 yrs, 12.9 m^3^/day; ≥81 yrs, 12.2 m^3^/day).

Korean adult males weighed more than Chinese, Japanese, and Nordic males, but Korean females weighed more than Chinese females [7, 10, 11]. In contrast, the average weights of Australians and Americans were suggested to be ~15 kg higher than that of Koreans [2, 9]. Because the BSA of each country was predicted using a calculation formula based on weight and height, the BSAs of Korea, China, and Japan were calculated to be smaller than those of Australia, Sweden, and the United States [2, 5, 9–11]. The NCD Risk Factor Collaboration [32] estimated trends in the average height for adults in 200 countries and reported that the most notable increase in height occurred in Korean women and Iranian men. Although Koreans are one of the tallest peoples among Asian countries [33], they are still different from Europeans, Americans and Australians due to their high protein intake and genetic factors, which are strongly correlated with tall statures [26].

The inhalation rate was calculated using various methods, such as double-labeled water, energy expenditure, and heart rate measurement [10, 12, 20, 34, 35]. The method of proposing a regression equation by simultaneously measuring heart rate and inhalation rate should consider factors that may affect the measured values, as emotional events, fever, and anxiety may increase the measurements of heart rate [36]. Inhalation rates may also be influenced by metabolic activity, body weight, health status, and other factors [4]. We predicted the long-term inhalation rate according to the US EPA method [19], which considers the daily activity pattern routinely performed on VE measurement data. The inhalation rate of Koreans was 14.61 m^3^/day, which was lower than those of Chinese (16.1 m^3^/day) and Japanese (17.3 m^3^/day) and higher than those of Australians (15 m^3^/day) and Europeans (15.2 m^3^/day for males and 11.3 m^3^/day for females). Since Wallis and Maconochie [37] suggested that the inhalation rate calculated from the United Kingdom may be applied to children in developing countries, the inhalation rate might be considered to have little correlation with socioeconomic and health status.

Food consumption is an important factor in assessing dietary exposure because it has a greater effect on the assessment of chemicals such as endocrine disruptors, pesticides, and pharmaceutical residues in foods and drinking water that are heavily consumed [38]. Owing to the similar food preferences among Asians, the grain and vegetable intakes in Koreans, Chinese, and Japanese accounted for a high proportion of total intake as shown in Table 4 [10, 11]. In addition, Koreans consumed 2.9–5.8 times more fish and seafood than other countries and about 30–50% less milk and dairy products than Australians and Americans [2, 7, 9–11].

**Table 4:** Recommended values of food and water ingestion rate in Korean and other countries’ exposure factor data.

Food group	Korea		China	Japan	Australia	Nordic	US
	(g/day)	(g/kg/day)	(g/day)	(g/day)	(g/day)	(g/day)	(g/kg/day)
Grains	600.23	9.59	402.1	303.5 (M) 233.3 (F)	340	214	2.1 (21–49 yrs) 1.7 (≥50 yrs)
Vegetables	425.82	6.76	325.3	435.4 (M) 404.1 (F)	260	274	2.5 (21–49 yrs) 2.6 (≥50 yrs)
Fruits	187.01	3.02	45.0	103.7 (M) 127.6 (F)	140	283	1.1 (21–49 yrs) 1.4 (≥50 yrs)
Meat products	116.69	1.83	73.9	98.9 (M) 72.3 (F)	160	132	1.8 (21–49 yrs) 1.4 (≥50 yrs)
Eggs	27.08	0.43	23.7	44.7 (M) 38.1 (F)	14	17	–
Fish and seafood	84.50	1.33	29.6	–	26	22	0.23 (21–49 yrs) 0.25 (≥50 yrs)
Nuts and seeds	6.49	0.10	–	–	5.1	–	–
Milk and dairy	85.06	1.38	26.5	112.7 (M) 122.8 (F)	290	–	3.2 (≥21 yrs)
Fats and oils	8.73	0.14	–	–	–	35	0.8–1.2
Sugars	10.18	0.16	–	–	–	34	–
Drinking water^a^	1008.33	15.93	1505	668 (M) 666 (F)	850	991–1201	858 (21–49 yrs) 902 (≥50 yrs)

^a^Drinking water: mL/day or mL/kg/day.

As people are continuously moving from one place to another, the time spent in particular places is crucial in assessing human exposure to environmental pollutants. The duration and frequency of exposure are determined by the time-activity pattern, or the activity pattern of an individual or population, and the time spent at each activity location [39, 40]. According to the existing exposure factor data, the time spent indoors by Koreans (1240 min) was the longest compared to other countries such as Australians (1200 min), Chinese (1167 min), and Americans (1142 min). On the other hand, Koreans spent half as much time outdoors as Americans and Chinese as shown in Table 5 [2, 9, 10]. Time spent indoors by Canadians (1379 min) was summed up including at home, away from home and in vehicle [8], and thus excluded from comparison with time-activity factors in other countries. Koreans spend more time indoors during the day than Americans, Australians, and Chinese, which might reflect the increased time of the internet at home and spending more time in indoors microenvironments such as restaurants, bars, shopping malls, cultural facilities and gyms considering the very high population density.

**Table 5:** Recommended values of time spent indoors, outdoors, and in transit in Korean and other countries’ exposure factor data (min/day).

Activity places	Korea	China	Australia	Canada	US
Indoors	1240	1167	1200	1379 (20–64 yrs) 1368 (≥64 yrs)	1142 (18–64 yrs) 1159 (≥65 yrs)
Outdoors	131	253	180	61 (20–64 yrs) 72 (≥64 yrs)	281 (18–64 yrs) 298 (≥65 yrs)
In transit	70	63	60	–	104.0 (18–64 yrs) 90.9 (≥65 yrs)

### Limitation

Formulas for calculating BSA using body weight and height have been reported in many studies [17, 41–44]. Among the previous studies, that of Lee et al. [17] was considered the most suitable formula for Koreans. However, there may be limitations in predicting the BSA for the elderly, with the results calculated for participants between the ages of 20 and 62. We calculated the daily inhalation rate by measuring each VE according to the intensity of physical activity using daily activity pattern data. However, because VE during sleep was difficult to measure under our laboratory conditions, the ratio of VE during sleep and VE at rest reported by the US EPA [20] was applied to estimate the VE during sleep in Koreans. In addition, the KNHANES used the 24 h recall method, which is a survey of the types, amounts, and recipes of all foods consumed by individuals in a short period to investigate the actual food intake. Although the survey was a key study with representative and reliable statistical data, it has limitations on the 24 h recall method, as memory errors in participants may occur and individual food intake varies widely daily. Lastly, the distinction between indoor and outdoor areas of individual activity was unclear in the time-use survey data from KOSTAT. For example, even if a participant reports that they have spent time at home, it may be outdoors, such as gardens and garages. Therefore, a small-scale (2526 people) survey was performed to calculate the indoor/outdoor time ratio of each place in this study, and by applying it, the 2014 Time-Use Survey data (28,152 people) was reclassified and reprocessed.

### Supplementary information

Supplementary Data

## Data Availability

Data from KNHANES and KOSTAT on life expectancy, body weight, BSA, and food and drinking water consumption were utilized to develop and update general exposure factors, which are important factors for exposure and risk assessment in Korean adults. Inhalation rates and time-activity patterns were derived through measurements and surveys, as well as existing national statistical data. These general exposure factors are expected to be utilized in various fields, such as human health risk assessment, epidemiological investigation, and the establishment of environmental standards. The Korean Exposure Factor Handbook contains technical information on the data sources used, the recommended values with statistical distributions, the methodologies for those values, their reliability and limitations, and comparison with resources from other countries that are available on the website of the National Institute of Environmental Research (NIER; https://ecolibrary.me.go.kr/nier/#/search/detail/5686028). Furthermore, this updated handbook provides detailed descriptions of exposure factors in Koreans categorized by gender, age group, general population, and doer-only (consumer-only) for various purposes. Developing and maintaining exposure factor resources is an ongoing challenge, given changes in food consumption patterns and anthropometric parameters over the past decade.
