# Supplementary material for: Updated general exposure factors for risk assessment in the Korean population
Source: J Expo Sci Environ Epidemiol. 2022 Apr 14;33(6):1013–20. doi: 10.1038/s41370-022-00437-6 (PMC10733140; doi:10.1038/s41370-022-00437-6)
Supplement: Supplementary file 1 — Supplementary Data [file 41370_2022_437_MOESM1_ESM.docx]

**Supplemental Data for**

Updated general exposure factors for risk assessment in the Korean population

Hyojung Yoon^1,3^, Jungkwan Seo^1,*^, Sun-Kyoung Yoo^1^, Pil-Je Kim^1^, Jinhyeon Park^2^, Youngtae Choe^2^, Wonho Yang^2,*^

1 Environmental Health Research Division, National Institute of Environmental Research, Incheon, Republic of Korea

2 Department of Occupational Health, Daegu Catholic University, Gyeongbuk, Republic of Korea

3 School of Environmental Engineering, University of Seoul, Seoul, Republic of Korea

* Corresponding authors: [whyang@cu.ac.kr](mailto:whyang@cu.ac.kr), [jkseo2001@korea.kr](mailto:jkseo2001@korea.kr)

**Contents:**

Table S1. Relative proportion (%) of each body part in general population of Korea.

Table S2. List of food groups and subgroups for intake calculation.

Table S3. Body weight (kg) according to sex and age groups.

Table S4. Body surface area (cm^2^) according to sex and age.

Table S5. Body surface area (cm^2^) for each body part according to the age group of Korean.

Table S6. Minute ventilation (L/min) measured in the laboratory.

Table S7. Daily inhalation rates (m^3^/day) according to sex

Table S8. Food and drinking water intake rate for doers (g/day).

Table S9. Time spent in indoor, outdoor and transportation by doers (h/day).

Table S10. Time-activity patterns at different locations according to sex (h/day).

.

Table S1. Relative proportion (%) of each body part in general population of Korea.

| Body Part | Head | Torso | Upper limbs | | | Lower limbs | | |
| --- | --- | --- | --- | --- | --- | --- | --- | --- |
|  |  |  | Arms | | Hands | Legs | | Feet |
|  |  |  | Upper | Fore |  | Thighs | Calves |  |
| Males | 7.5 | 37.3 | 8.7 | 6.2 | 4.9 | 14.9 | 13.7 | 6.8 |
| Females | 7.8 | 37.5 | 8.8 | 5.9 | 4.8 | 15.6 | 13.6 | 6.1 |
| Both sexes combined | 7.6 | 37.4 | 8.8 | 6 | 4.8 | 15.3 | 13.6 | 6.5 |

Table S2. List of food groups and subgroups for intake calculation.

| Food Groups  (14) | Subgroups  (150) | Criteria for intake calculation |
| --- | --- | --- |
| Grain products  (11) | Processed grains, grain powder, noodles, rice cake, ramyeon, bread, snacks, cereal, steamed rice, corn, multigrain steamed rice | Foods, ingredients |
| Vegetables  (28) | Eggplant, potato, sweet potato, hot pepper, sesame leaves, carrot, tofu, garlic, radish, water dropwort, Napa cabbage, mushrooms, broccoli, lettuce, mungbean/bean sprouts, spinach, mugwort, cabbage, onion, cucumber, ginseng, beans, tomato, green onion/ Chinese chive, adzuki beans, sweet pepper/ paprika, pumpkin, other vegetables | Ingredients |
| Fruits  (14) | Persimmon, mandarin orange, strawberry, banana, pear, peach, blueberry, apple, watermelon, orange, oriental melon, pineapple, grape, other fruits | Ingredients |
| Meat products  (6) | Chicken, pork, by-products, beef, processed meats, Other meats | Ingredients |
| Eggs  (3) | Chicken egg, quail egg, Other eggs | Ingredients |
| Fish and shellfish  (20) | Skipjack tuna/tuna, hair tail, crab, mackerel, oyster, Pacific saury, long-arm octopus, cod, anchovy, Alaska pollack, octopus/squid, little neck clam, shrimp, liquid salt-fermented fish, fish paste, yellow croaker, hard-shelled mussel, other fish, other shellfish, other seafood | Ingredients |
| Nuts and seeds products  (7) | Sesame, peanut, chestnut, almond, ginkgo nut, walnut, Other nuts and seeds | Ingredients |
| Seaweeds  (5) | Laver, sea tangle, sea mustard, sea lettuce, Other seaweeds | Raw, dried |
| Milk and dairy products  (8) | Ice cream, liquid-type yogurt, milk, modified milk, cheese, curd-type yogurt, other milk and dairy products | Foods |
| Fats and Oils  (8) | Margarine, butter, corn oil, olive oil, sesame oil, coffee creamer, soybean oil, Other fats and oils | Foods |
| Beverages  (10) | Fruit beverages, functional beverages, soybean milk, isotonic drink, alcoholic beverages, teas, vegetable beverages, coffee beverages, cocoa, carbonated beverages | Foods |
| Seasonings  (12) | Soy sauce, fermented red pepper paste, sesame seeds, soybean paste/ mixed soybean paste with red pepper paste, mayonnaise, salad dressing, salt, sauces, vinegar, ketchup, pepper, other seasonings | Foods |
| Sugars and sweeteners (10) | Gum, honey, candy, sugar, syrup/starch syrup, jelly, jam, chocolate, caramel, Other sugars | Foods |
| Fabricated foods  (8) | Dumpling, fried rice, sandwich, rice gruel, pizza, hotdog, hamburger, Other fabricated foods | Foods |

**Table S3**. Body weight (kg) according to sex and age groups.

| Sex | Age group  (years) | N | Mean* | S.D.** | 5th | 25th | 50th | 75th | 95th |
| --- | --- | --- | --- | --- | --- | --- | --- | --- | --- |
| Male | 19–24 | 683 | 70.7 | 12.60 | 54.5 | 62.7 | 69.3 | 77.4 | 97.2 |
|  | 25–34 | 1,273 | 75.6 | 12.72 | 58.5 | 67.0 | 74.4 | 83.1 | 98.3 |
|  | 35–44 | 1,870 | 75.2 | 11.84 | 58.1 | 66.9 | 74.1 | 81.6 | 95.8 |
|  | 45–54 | 1,736 | 71.4 | 10.10 | 55.9 | 64.8 | 70.7 | 77.7 | 88.6 |
|  | 55–64 | 1,837 | 68.6 | 9.55 | 53.6 | 62.2 | 68.2 | 74.4 | 85.4 |
|  | 65–74 | 1,626 | 65.6 | 9.33 | 51.0 | 59.0 | 65.7 | 71.9 | 81.1 |
|  | ≥75 | 925 | 61.7 | 9.01 | 46.8 | 55.3 | 61.4 | 67.6 | 76.3 |
|  | Total | 9,950 | 71.5 | 11.74 | 53.1 | 62.6 | 69.3 | 76.8 | 90.3 |
| Female | 19–24 | 842 | 56.0 | 9.77 | 43.7 | 49.9 | 54.4 | 60.5 | 75.7 |
|  | 25–34 | 1,691 | 57.7 | 10.54 | 44.9 | 50.7 | 55.8 | 62.6 | 77.9 |
|  | 35–44 | 2,422 | 58.5 | 9.58 | 46.0 | 51.9 | 56.8 | 63.5 | 76.0 |
|  | 45–54 | 2,472 | 59.0 | 8.69 | 46.7 | 52.9 | 57.8 | 63.6 | 74.2 |
|  | 55–64 | 2,433 | 58.4 | 8.53 | 46.6 | 52.7 | 57.6 | 63.7 | 74.0 |
|  | 65–74 | 1,999 | 57.6 | 8.32 | 44.2 | 51.9 | 56.9 | 62.3 | 71.5 |
|  | ≥75 | 1,358 | 53.4 | 9.02 | 39.6 | 46.7 | 53.0 | 59.2 | 68.8 |
|  | Total | 13,217 | 57.7 | 9.39 | 44.6 | 51.5 | 56.6 | 62.8 | 74.1 |
| Both sexes combined | 19–24 | 1,525 | 63.8 | 13.52 | 45.4 | 53.0 | 60.6 | 70.6 | 88.2 |
|  | 25–34 | 2,964 | 67.0 | 14.73 | 46.2 | 54.3 | 63.2 | 74.5 | 92.1 |
|  | 35–44 | 4,292 | 67.0 | 13.63 | 47.4 | 55.5 | 63.7 | 74.4 | 89.8 |
|  | 45–54 | 4,208 | 65.3 | 11.28 | 48.4 | 55.8 | 62.9 | 71.2 | 83.9 |
|  | 55–64 | 4,270 | 63.4 | 10.37 | 47.7 | 55.3 | 62.2 | 69.5 | 81.5 |
|  | 65–74 | 3,625 | 61.3 | 9.67 | 46.1 | 54.1 | 60.4 | 67.4 | 77.8 |
|  | ≥75 | 2,283 | 56.4 | 9.85 | 41.2 | 49.5 | 56.4 | 63.1 | 73.5 |
|  | Total | 23,167 | 64.5 | 12.65 | 46.3 | 54.4 | 61.6 | 70.3 | 84.8 |

* Arithmetic mean; ** S.D.: standard deviation

**Table S4.** Body surface area (cm^2^) according to sex and age.

| Sex | Age group  (years) | Mean* | S.D.** | 5th | 25th | 50th | 75th | 95th |
| --- | --- | --- | --- | --- | --- | --- | --- | --- |
| Male | 19–24 | 18,811 | 1,584 | 16,435 | 17,801 | 18,752 | 19,814 | 21,627 |
|  | 25–34 | 19,418 | 1,615 | 17,045 | 18,317 | 19,325 | 20,457 | 22,222 |
|  | 35–44 | 19,245 | 1,544 | 16,904 | 18,146 | 19,132 | 20,172 | 21,915 |
|  | 45–54 | 18,615 | 1,402 | 16,351 | 17,727 | 18,556 | 19,535 | 20,945 |
|  | 55–64 | 18,107 | 1,367 | 15,850 | 17,189 | 18,088 | 18,981 | 20,426 |
|  | 65–74 | 17,616 | 1,333 | 15,494 | 16,681 | 17,628 | 18,517 | 19,836 |
|  | ≥75 | 16,973 | 1,306 | 14,821 | 16,057 | 16,957 | 17,853 | 19,105 |
|  | Total | 18,670 | 1,625 | 15,842 | 17,357 | 18,370 | 19,479 | 21,221 |
| Female | 19–24 | 16,163 | 1,360 | 14,249 | 15,285 | 16,015 | 16,890 | 18,722 |
|  | 25–34 | 16,329 | 1,403 | 14,307 | 15,375 | 16,175 | 17,126 | 18,779 |
|  | 35–44 | 16,334 | 1,297 | 14,455 | 15,411 | 16,195 | 17,119 | 18,592 |
|  | 45–54 | 16,228 | 1,199 | 14,365 | 15,404 | 16,152 | 16,926 | 18,312 |
|  | 55–64 | 15,985 | 1,175 | 14,220 | 15,192 | 15,952 | 16,752 | 18,072 |
|  | 65–74 | 15,695 | 1,213 | 13,635 | 14,877 | 15,654 | 16,452 | 17,640 |
|  | ≥75 | 14,911 | 1,341 | 12,803 | 13,983 | 14,899 | 15,795 | 17,133 |
|  | Total | 16,058 | 1,339 | 13,896 | 15,130 | 15,937 | 16,797 | 18,249 |
| Both sexes combined | 19–24 | 17,561 | 1,987 | 14,573 | 15,860 | 17,158 | 18,760 | 20,927 |
|  | 25–34 | 17,938 | 2,164 | 14,630 | 15,956 | 17,432 | 19,179 | 21,390 |
|  | 35–44 | 17,817 | 2,039 | 14,757 | 16,001 | 17,401 | 19,010 | 21,075 |
|  | 45–54 | 17,436 | 1,769 | 14,689 | 15,892 | 17,006 | 18,436 | 20,260 |
|  | 55–64 | 17,031 | 1,657 | 14,454 | 15,690 | 16,766 | 18,053 | 19,804 |
|  | 65–74 | 16,580 | 1,590 | 14,039 | 15,416 | 16,442 | 17,647 | 19,290 |
|  | ≥75 | 15,647 | 1,656 | 13,086 | 14,557 | 15,701 | 16,874 | 18,561 |
|  | Total | 17,352 | 1,979 | 14,259 | 15,663 | 16,828 | 18,296 | 20,418 |

* Arithmetic mean; ** S.D.: standard deviation

Table S5. Body surface area (cm^2^) for each body part according to the age group of Korean.

| Age group | Body Part | Mean* | S.D.** | 5th | 25th | 50th | 75th | 95th |
| --- | --- | --- | --- | --- | --- | --- | --- | --- |
| 19–24 | Body | 17,561 | 1,987 | 14,573 | 15,860 | 17,158 | 18,760 | 20,927 |
|  | Head | 1,340 | 136 | 1,136 | 1,233 | 1,317 | 1,417 | 1,575 |
|  | Torso | 6,566 | 731 | 5,465 | 5,946 | 6,417 | 7,003 | 7,806 |
|  | Arms | 2,601 | 306 | 2,142 | 2,334 | 2,538 | 2,790 | 3,116 |
|  | Hands | 853 | 102 | 700 | 763 | 830 | 915 | 1,025 |
|  | Legs | 5,061 | 544 | 4,239 | 4,612 | 4,959 | 5,377 | 5,992 |
|  | Thighs | 2,670 | 265 | 2,271 | 2,460 | 2,627 | 2,824 | 3,130 |
|  | Calves | 2,391 | 282 | 1,967 | 2,144 | 2,331 | 2,564 | 2,865 |
|  | Feet | 1,141 | 176 | 889 | 971 | 1,083 | 1,263 | 1,420 |
| 25–34 | Body | 17,938 | 2,164 | 14,630 | 15,956 | 17,432 | 19,179 | 21,390 |
|  | Head | 1,369 | 147 | 1,141 | 1,243 | 1,338 | 1,447 | 1,612 |
|  | Torso | 6,706 | 797 | 5,486 | 5,983 | 6,521 | 7,158 | 7,979 |
|  | Arms | 2,657 | 333 | 2,151 | 2,347 | 2,569 | 2,853 | 3,184 |
|  | Hands | 871 | 112 | 702 | 766 | 840 | 938 | 1,046 |
|  | Legs | 5,169 | 593 | 4,255 | 4,641 | 5,035 | 5,500 | 6,120 |
|  | Thighs | 2,728 | 288 | 2,279 | 2,483 | 2,670 | 2,880 | 3,208 |
|  | Calves | 2,442 | 308 | 1,975 | 2,156 | 2,360 | 2,623 | 2,927 |
|  | Feet | 1,165 | 190 | 892 | 975 | 1,085 | 1,295 | 1,451 |
| 35–44 | Body | 17,817 | 2,039 | 14,757 | 16,001 | 17,401 | 19,010 | 21,075 |
|  | Head | 1,360 | 138 | 1,151 | 1,244 | 1,336 | 1,436 | 1,584 |
|  | Torso | 6,662 | 750 | 5,533 | 5,999 | 6,511 | 7,095 | 7,864 |
|  | Arms | 2,639 | 315 | 2,169 | 2,352 | 2,569 | 2,829 | 3,140 |
|  | Hands | 865 | 106 | 708 | 768 | 840 | 930 | 1,032 |
|  | Legs | 5,136 | 557 | 4,293 | 4,651 | 5,030 | 5,452 | 6,031 |
|  | Thighs | 2,711 | 269 | 2,301 | 2,487 | 2,665 | 2,859 | 3,150 |
|  | Calves | 2,425 | 290 | 1,992 | 2,160 | 2,360 | 2,601 | 2,887 |
|  | Feet | 1,155 | 182 | 901 | 977 | 1,089 | 1,284 | 1,432 |
| 45–54 | Body | 17,436 | 1,769 | 14,689 | 15,892 | 17,006 | 18,436 | 20,260 |
|  | Head | 1,332 | 119 | 1,143 | 1,234 | 1,310 | 1,394 | 1,526 |
|  | Torso | 6,520 | 650 | 5,508 | 5,959 | 6,367 | 6,881 | 7,558 |
|  | Arms | 2,582 | 274 | 2,160 | 2,339 | 2,509 | 2,739 | 3,018 |
|  | Hands | 846 | 92 | 705 | 764 | 821 | 900 | 992 |
|  | Legs | 5,027 | 482 | 4,273 | 4,619 | 4,919 | 5,288 | 5,798 |
|  | Thighs | 2,654 | 232 | 2,284 | 2,467 | 2,615 | 2,778 | 3,033 |
|  | Calves | 2,373 | 253 | 1,983 | 2,148 | 2,304 | 2,518 | 2,775 |
|  | Feet | 1,130 | 162 | 896 | 972 | 1,059 | 1,240 | 1,377 |
| 55–64 | Body | 17,031 | 1,657 | 14,454 | 15,690 | 16,766 | 18,053 | 19,804 |
|  | Head | 1,302 | 112 | 1,124 | 1,217 | 1,290 | 1,369 | 1,490 |
|  | Torso | 6,369 | 609 | 5,420 | 5,881 | 6,278 | 6,739 | 7,387 |
|  | Arms | 2,521 | 257 | 2,126 | 2,310 | 2,475 | 2,682 | 2,949 |
|  | Hands | 826 | 86 | 694 | 755 | 810 | 881 | 969 |
|  | Legs | 4,911 | 452 | 4,204 | 4,557 | 4,853 | 5,184 | 5,665 |
|  | Thighs | 2,594 | 219 | 2,248 | 2,431 | 2,574 | 2,725 | 2,960 |
|  | Calves | 2,317 | 237 | 1,952 | 2,122 | 2,273 | 2,465 | 2,711 |
|  | Feet | 1,101 | 153 | 883 | 962 | 1,050 | 1,214 | 1,345 |
| 65–74 | Body | 16,580 | 1,590 | 14,039 | 15,416 | 16,442 | 17,647 | 19,290 |
|  | Head | 1,269 | 108 | 1,089 | 1,193 | 1,263 | 1,340 | 1,452 |
|  | Torso | 6,201 | 585 | 5,260 | 5,776 | 6,154 | 6,593 | 7,198 |
|  | Arms | 2,453 | 246 | 2,065 | 2,269 | 2,430 | 2,621 | 2,874 |
|  | Hands | 804 | 82 | 674 | 742 | 795 | 861 | 945 |
|  | Legs | 4,784 | 435 | 4,077 | 4,474 | 4,753 | 5,070 | 5,522 |
|  | Thighs | 2,530 | 213 | 2,178 | 2,381 | 2,518 | 2,668 | 2,886 |
|  | Calves | 2,255 | 227 | 1,897 | 2,084 | 2,232 | 2,409 | 2,642 |
|  | Feet | 1,068 | 145 | 857 | 948 | 1,042 | 1,186 | 1,310 |
| ≥75 | Body | 15,647 | 1,656 | 13,086 | 14,557 | 15,701 | 16,874 | 18,561 |
|  | Head | 1,202 | 115 | 1,018 | 1,129 | 1,208 | 1,287 | 1,398 |
|  | Torso | 5,856 | 611 | 4,907 | 5,458 | 5,882 | 6,305 | 6,923 |
|  | Arms | 2,312 | 254 | 1,924 | 2,142 | 2,319 | 2,507 | 2,765 |
|  | Hands | 757 | 85 | 628 | 700 | 758 | 823 | 908 |
|  | Legs | 4,523 | 457 | 3,800 | 4,228 | 4,546 | 4,863 | 5,318 |
|  | Thighs | 2,399 | 226 | 2,036 | 2,254 | 2,409 | 2,564 | 2,782 |
|  | Calves | 2,124 | 234 | 1,767 | 1,968 | 2,130 | 2,304 | 2,542 |
|  | Feet | 997 | 144 | 798 | 893 | 983 | 1,125 | 1,260 |

* Arithmetic mean; ** S.D.: standard deviation

**Table S6.** Minute ventilation (L/min) measured in the laboratory.

| Sex | Activity level | Mean | 5th | 25th | 50th | 75th | 95th |
| --- | --- | --- | --- | --- | --- | --- | --- |
| Male  (n= 97) | Sleep^*^ | 6.6 | 5.3 | 6 | 6.6 | 7.1 | 8.1 |
|  | Rest | 7.1 | 5.7 | 6.5 | 7.1 | 7.7 | 8.7 |
|  | Slow walking | 18.1 | 13.8 | 16.3 | 18 | 19.9 | 22.3 |
|  | Fast walking | 33.0 | 26 | 29.9 | 32.4 | 35.5 | 40.5 |
| Female  (n= 97) | Sleep^*^ | 5.6 | 3.9 | 4.8 | 5.6 | 6.1 | 7.2 |
|  | Rest | 6.0 | 4.2 | 5.2 | 6.1 | 6.6 | 7.8 |
|  | Slow walking | 14.2 | 11 | 12.6 | 14.1 | 15.6 | 18.4 |
|  | Fast walking | 25.3 | 19.5 | 22 | 25.5 | 27.9 | 31.5 |

* The VE during sleep for Koreans was estimated by applying the ratio of VE during sleep to VE at rest, as reported by the US Environmental Protection Agency (US EPA, 2009).

**Table S7.** Daily inhalation rates (m^3^/day) according to sex.

| Sex | Mean* | S.D.** | 5th | 25th | 50th | 75th | 95th |
| --- | --- | --- | --- | --- | --- | --- | --- |
| Male | 16.2 | 2.9 | 11.4 | 14.0 | 16.4 | 18.3 | 21.0 |
| Female | 13.0 | 2.6 | 9.2 | 11.1 | 13.3 | 14.9 | 16.9 |
| Both sexes combined | 14.6 | 3.2 | 9.7 | 12.3 | 14.7 | 16.6 | 20.2 |

* Arithmetic mean; ** S.D.: standard deviation

**Table S8**. Food and drinking water intake rate for doers (g/day).

| Food groups | | N | Frequency  (%) | Mean* | S.D.** | 25th | 50th | 75th | 95th |
| --- | --- | --- | --- | --- | --- | --- | --- | --- | --- |
| Grains | Foods | 31,290 | 100 | 603.26 | 315.89 | 387.88 | 560.21 | 761.88 | 1161.73 |
|  | Ingredients | 31,224 | 100 | 242.37 | 125.76 | 157.55 | 227.34 | 307.94 | 455.78 |
| Vegetables | | 31,290 | 100 | 428.11 | 299.80 | 227.67 | 365.95 | 552.67 | 966.56 |
| Fruits | | 21,317 | 100 | 284.08 | 331.90 | 71.20 | 192.15 | 384.32 | 889.82 |
| Meat products | | 21,952 | 100 | 157.47 | 241.70 | 36.52 | 87.63 | 186.44 | 518.07 |
| Eggs | | 15,823 | 100 | 49.74 | 52.24 | 13.50 | 33.72 | 66.87 | 143.26 |
| Fish and seafood | | 26,612 | 100 | 99.61 | 173.13 | 11.11 | 45.55 | 122.50 | 368.75 |
| Nuts and seeds products | | 23,841 | 100 | 8.52 | 40.54 | 0.30 | 0.96 | 3.96 | 37.66 |
| Seaweeds | Raw | 15,435 | 100 | 46.66 | 87.77 | 13.84 | 29.00 | 55.89 | 137.48 |
|  | Dried | 15,435 | 100 | 5.51 | 12.87 | 1.52 | 3.25 | 6.49 | 16.00 |
| Milk and dairy products | | 11,938 | 100 | 215.70 | 178.41 | 85.00 | 201.90 | 278.00 | 540.60 |
| Fats and oils | | 28,181 | 100 | 9.57 | 11.82 | 2.09 | 5.94 | 12.91 | 30.71 |
| Beverages**^*^** | | 25,479 | 100 | 332.18 | 585.08 | 12.29 | 134.66 | 406.35 | 1335.78 |
| Seasonings | | 30,928 | 100 | 62.49 | 75.60 | 19.66 | 42.84 | 79.70 | 180.66 |
| Sugars and sweeteners | | 24,433 | 100 | 12.79 | 21.61 | 1.93 | 6.38 | 15.61 | 45.10 |
| Fabricated foods | | 1,870 | 100 | 123.20 | 119.73 | 37.95 | 88.71 | 181.77 | 345.07 |
| Water*** | | 31,263 | 100 | 1012.10 | 655.37 | 600 | 1000 | 1200 | 2000 |

* Arithmetic mean; ** S.D.: standard deviation

** Beverages and drinking water were calculated in mL/day.

**Table S9**. Time spent in indoor, outdoor and transportation by doers (h/day).

| Category | Location | | N | Frequency  (%) | Mean* | S.D.** | 25th | 50th | 75th | 95th |
| --- | --- | --- | --- | --- | --- | --- | --- | --- | --- | --- |
| Weekdays | Indoors | House | 28,037 | 99.59 | 15.16 | 4.67 | 11.49 | 14.16 | 19.15 | 23.15 |
|  |  | Workplace | 14,517 | 51.57 | 7.42 | 3.17 | 5.57 | 8.06 | 9.44 | 11.84 |
|  |  | School | 1,065 | 3.78 | 4.77 | 3.15 | 2.38 | 4.77 | 6.92 | 9.95 |
|  |  | Others | 23,806 | 84.56 | 1.95 | 1.91 | 0.66 | 1.38 | 2.66 | 5.45 |
|  | Outdoors | | 28,107 | 99.84 | 2.07 | 1.93 | 0.79 | 1.52 | 2.74 | 5.80 |
|  | Transport | | 19,839 | 70.47 | 1.66 | 1.10 | 1.00 | 1.33 | 2.00 | 3.67 |
| Weekends | Indoors | House | 18,723 | 99.59 | 17.07 | 4.61 | 13.6 | 17.61 | 20.82 | 23.92 |
|  |  | Workplace | 4,566 | 24.29 | 6.58 | 3.61 | 3.83 | 7.09 | 9.12 | 12.00 |
|  |  | School | 218 | 1.16 | 2.45 | 2.59 | 0.52 | 1.58 | 3.08 | 8.01 |
|  |  | Others | 16,257 | 86.47 | 2.17 | 1.85 | 0.83 | 1.70 | 2.98 | 5.78 |
|  | Outdoors | | 18,791 | 99.95 | 2.34 | 2.12 | 0.92 | 1.83 | 3.15 | 6.50 |
|  | Transport | | 12,512 | 66.55 | 1.74 | 1.29 | 0.83 | 1.33 | 2.17 | 4.33 |
| Both | Indoors | House | 46,760 | 99.59 | 15.93 | 4.74 | 12.12 | 15.77 | 19.99 | 23.5 |
|  |  | Workplace | 19,805 | 42.18 | 7.24 | 3.27 | 5.18 | 7.86 | 9.36 | 11.87 |
|  |  | School | 1,283 | 2.73 | 4.38 | 3.18 | 1.57 | 4.31 | 6.66 | 9.72 |
|  |  | Others | 40,063 | 85.33 | 2.04 | 1.89 | 0.72 | 1.5 | 2.79 | 5.59 |
|  | Outdoors | | 46,898 | 99.88 | 2.18 | 2.01 | 0.83 | 1.64 | 2.91 | 6.09 |
|  | Transport | | 32,351 | 68.9 | 1.69 | 1.18 | 1.00 | 1.33 | 2.17 | 4.00 |

* Arithmetic mean; ** S.D.: standard deviation

Table S10. Time-activity patterns at different locations according to sex (h/day).

| Category | Location | | Male | | Female | |
| --- | --- | --- | --- | --- | --- | --- |
|  |  |  | Mean* | S.D.** | Mean* | S.D. |
| Weekdays  (male: 13,018, female: 15,134) | Indoors | House | 13.41 | 4.45 | 16.56 | 4.53 |
|  |  | Workplace | 4.70 | 4.44 | 3.08 | 4.13 |
|  |  | School | 0.22 | 1.22 | 0.15 | 0.97 |
|  |  | Restaurant | 0.56 | 0.82 | 0.35 | 0.72 |
|  |  | Bar | 0.10 | 0.17 | 0.02 | 0.05 |
|  |  | Shopping mall/Market | 0.06 | 0.12 | 0.23 | 0.35 |
|  |  | Cultural facility | 0.05 | 0.2 | 0.06 | 0.10 |
|  |  | Gym | 0.23 | 0.47 | 0.15 | 0.26 |
|  |  | Others | 0.86 | 1.29 | 0.69 | 1.19 |
|  | Outdoors | - | 2.36 | 2.18 | 1.83 | 1.65 |
|  | Transport | Bicycle | 0.03 | 0.20 | 0.01 | 0.10 |
|  |  | Private vehicles | 0.94 | 1.19 | 0.43 | 0.82 |
|  |  | Public | 0.34 | 0.8 | 0.41 | 0.79 |
|  |  | Others | 0.15 | 0.57 | 0.07 | 0.34 |
| Weekends  (male: 8,706, female: 10,094) | Indoors | House | 15.93 | 4.85 | 17.92 | 4.41 |
|  |  | Workplace | 1.91 | 3.46 | 1.33 | 3.20 |
|  |  | School | 0.04 | 0.48 | 0.02 | 0.28 |
|  |  | Restaurant | 0.57 | 0.93 | 0.41 | 0.83 |
|  |  | Bar | 0.10 | 0.21 | 0.03 | 0.06 |
|  |  | Shopping mall/Market | 0.21 | 0.27 | 0.38 | 0.50 |
|  |  | Cultural facility | 0.18 | 0.38 | 0.25 | 0.43 |
|  |  | Gym | 0.07 | 0.10 | 0.04 | 0.07 |
|  |  | Others | 0.64 | 0.83 | 0.87 | 1.03 |
|  | Outdoors | - | 3.03 | 2.48 | 1.74 | 1.51 |
|  | Transport | Bicycle | 0.03 | 0.19 | 0.01 | 0.08 |
|  |  | Private vehicles | 0.93 | 1.21 | 0.61 | 1.05 |
|  |  | Public | 0.26 | 0.79 | 0.32 | 0.8 |
|  |  | Others | 0.10 | 0.53 | 0.07 | 0.46 |

* Arithmetic mean; ** S.D.: standard deviation
